# Statistical Analysis and Predictive Modelling of Chip Temperature in the Cutting Zone for Tools with Different Rake Angles

**DOI:** 10.3390/ma19214728

**Published:** 2026-11-06

**Authors:** Ireneusz Zagórski, Monika Kulisz, Agnieszka Skoczylas, Piotr Zgórniak, Stanisław Legutko

**Affiliations:** 1 Department of Production Engineering, Faculty of Mechanical Engineering, Lublin University of Technology, Nadbystrzycka 36, 20-618 Lublin, Poland; a.skoczylas@pollub.pl; 2 Department of Organisation of Enterprise, Faculty of Management, Lublin University of Technology, 20-618 Lublin, Poland; m.kulisz@pollub.pl; 3 Institute of Machine Tools and Production Engineering, Faculty of Mechanical Engineering, Lodz University of Technology, Stefanowskiego 1/15, 90-537 Łódź, Poland; piotr.zgorniak@p.lodz.pl; 4 Institute of Mechanical Technology, Faculty of Mechanical Engineering, Poznan University of Technology, Piotrowo 2, 61-138 Poznań, Poland; stanislaw.legutko@put.poznan.pl

**Keywords:** magnesium alloys, rough milling, temperature in cutting zone, ANOVA analysis, rake angle, modeling

## Abstract

Experimental thermographic data from dry rough milling of AZ31B and AZ91D magnesium alloys using tools with rake angles of 5° and 30° were reanalysed using descriptive statistics and predictive modelling. Depending on the assumptions of normality and homogeneity of variance, a one-way ANOVA, Welch ANOVA or Kruskal–Wallis test was applied to up to ten highest available readings of each run; as these are repeated measurements within a run, the tests are interpreted descriptively and supplemented with effect sizes. Descriptive comparisons showed differences in the selected upper-temperature readings across parameter levels within the alloy–rake-angle groups. To provide a consistently defined upper-tail response, whose estimation precision depends on the number of recorded frames, the 95th percentile (T95) of the thermographic observations was adopted as the predictive response. Gaussian process regression (GPR) was evaluated using leave-one-out cross-validation and a clipped jackknife+-type procedure. The cutting-speed paths had the lowest mean absolute prediction error (9.7 °C), compared with 17.7 °C for feed per tooth and 18.7 °C for axial depth of cut. The nominal 95% GPR prediction intervals achieved only 63.4% mean empirical coverage, increasing to 85.2% with the clipped procedure, which has no standard coverage guarantee and remains below the nominal level. Linear and quadratic regression provided comparable point-prediction accuracy and, with the same clipped procedure, comparable coverage (85.2% and 86.6%), whereas a shallow neural network performed worse. Increased model complexity did not consistently improve prediction in this dataset. These results demonstrate that assessment of chip-temperature models requires out-of-sample validation and explicit calibration of predictive uncertainty.

## 1. Introduction

Temperature is a key physical quantity characterizing machining processes, as it affects tool wear and tool life, dimensional accuracy, surface integrity, and the thermal state of the workpiece. These aspects are especially important in milling due to the intermittent nature of the process and transient thermal loading, which complicates both temperature measurement and prediction. In the machining of magnesium alloys, temperature is important also from the perspective of process safety [1]. Magnesium alloys are lightweight engineering materials with favorable specific properties, but chips and fine particles may ignite under unfavorable conditions [2,3,4]. The ignition risk depends on cutting conditions, including cutting speed, feed rate, and depth of cut [4]. Tool geometry, and in particular the tool’s rake angle, affects chip formation, contact conditions, cutting forces, and heat generation. Numerical and experimental studies indicate that the relationship between rake angle and temperature is not necessarily monotonic and that thermal effects depend on the overall cutting conditions [5].

The temperature during the milling process can be influenced by many factors, in particular by cutting speed. When machining magnesium alloys, it is crucial to maintain the machining conditions in such a way as to achieve high milling efficiency without generating excessive heat in the chip, as this may increase the risk of chip ignition and, in extreme cases, fire. The geometry of the cutting tool, and in particular the rake angle, plays a significant role in the chip formation process. Numerous literature sources have analyzed the effect of rake angle on cutting temperature [6,7,8]. In many machining conditions, increasing the positive rake angle reduces cutting forces and heat generation; however, the resulting thermal response is not necessarily monotonic and depends on the workpiece material and cutting parameters. The rake angle can significantly affect the temperature of the chip formed, as it alters the geometry of material deformation, the cutting forces, the contact length between the chip and the rake face, and the contribution of friction to the energy balance. Typically, increasing a positive rake angle may lead to a reduction in chip temperature, but this relationship need not be monotonic across the entire range of cutting parameters.

The power consumed in deforming the material depends mainly on the cutting speed and the shear force, and a significant proportion of it is converted into heat. As the positive rake angle increases, the material is generally subjected to less intense compression in front of the cutting edge. The specific energy required to form a unit volume of chip decreases. Once formed, the chip must move along the rake face. A larger positive rake angle usually facilitates chip flow and can reduce both the contact load and the length of contact between the chip and the rake face. As a result, the area of intense friction may be reduced [5]. The geometry of the chip also changes. The rake angle affects the chip ratio. With a less favorable cutting-edge geometry, the workpiece is compressed more strongly, so the chip may be more compressed. With a larger positive rake angle, the deformation of the material may be lower and the chip may be thinner.

This has an interesting consequence: the temperature and the total thermal energy of the chip are not the same. Therefore, a high temperature in a thin section of the chip does not necessarily mean that it carries more total energy than a thicker chip at a lower temperature. At high cutting speeds, the chip plays a very important role, as it quickly leaves the cutting zone and transports energy out of the system. It cannot therefore be assumed that increasing the rake angle will reduce friction and, consequently, always result in a lower chip temperature. The mechanism is more complex, as deformation, chip geometry, contact time, contact area, and heat distribution between the chip, tool, and workpiece all change simultaneously. Studies of heat distribution show that the geometry of the cutting edge and the thickness of the cut layer can significantly alter this distribution [5]. Protective coatings applied to the cutting edges of cutting tools are also a significant factor influencing the heat balance in the cutting process. They modify heat partition between the chip, cutting tool, and workpiece. In high-speed machining, a considerable proportion of the generated heat may be transported away with the chip. Detailed studies on heat flow and temperature distribution for coated cutting tools can be found in [9,10,11].

When the rake angle is changed, the following parameters may change simultaneously: the shear angle, chip ratio, chip–tool contact length, cutting forces, contact stresses, friction, contact time, local chip-flow velocity, heat distribution, and the geometry and mass of the observed chip fragment. Furthermore, milling is a discontinuous process. The thickness of the machined layer varies as the cutting edge rotates, and the cutting edge cyclically passes through heating and cooling phases. The size of the chip is of particular importance, especially during precision milling with small cutting depths and low feed rates at high cutting speeds [12].

Thermal imaging measurements of the temperature of small, rapidly moving chips are particularly difficult because of the spatial and temporal resolution limitations of infrared cameras. The further the camera is from the object, the more difficult it becomes to reliably detect very small chip regions. High-speed cameras can therefore be used to complement thermal measurements by recording chip formation and the behavior of small chips in the cutting zone [13]. In thermal imaging measurements, microscopic lenses may be used to narrow the camera field of view by positioning the optical system close to the cutting zone. Such measurements are widely used in orthogonal cutting models and commonly involve measurement of the temperature of the tool, chip, and chip-formation zone [14,15,16]. Turning and model intermittent cutting have also been investigated to provide insight into transient thermal phenomena relevant to milling [17,18,19].

Therefore, thermal models for milling must take into account temperature variations over time. A very important aspect is the selection of appropriate thermal imaging acquisition parameters in order to obtain reliable measurement results [20]. It is particularly important to use an appropriate frame rate while taking into account the integration time and cutting parameters, as well as the influence of the number of cutting edges, the cross-sectional dimensions of the chip, the cutting-tool material, specimen dimensions, frame resolution, emissivity, and the distance between the detector and the cutting zone [21,22].

Experimental results for the milling of magnesium alloys show precisely these challenges. The direct basis for the present study is the experimental work [23], which measured chip temperature during dry rough milling of AZ31B and AZ91D magnesium alloys using tools with rake angles of γ = 5° and γ = 30°. While that study provided valuable experimental data, its analysis was primarily descriptive and did not establish predictive performance. The aim of the present study is therefore to transform this experimental dataset into a framework for descriptive statistical comparison and run-level predictive evaluation. Gaussian process regression is adopted to model chip temperature because it provides a flexible non-parametric model together with predictive uncertainty estimates. Predictive performance is evaluated using leave-one-out cross-validation, and uncertainty is further assessed using a clipped jackknife+-type procedure [24,25,26].

The companion study [23] established, through systematic thermographic measurements, that chip temperature during dry rough milling of AZ31B and AZ91D remained below the ignition threshold considered there (an apparent surface temperature, which by itself does not demonstrate negligible ignition risk; see Section 4) across the investigated ranges of cutting speed, feed per tooth, axial depth of cut, and rake angle. The relationships were characterized using box plots, bar charts, and second-degree polynomial trend lines fitted separately to the twelve technological paths. This descriptive approach, however, leaves three problems unresolved. First, the trend lines report goodness-of-fit (R^2^) but not predictive accuracy under held-out validation, so it is not established whether they generalize beyond the specific points used to fit them. Second, no uncertainty is attached to the resulting temperature estimates: a point prediction of, for example, 320 °C provides no information about how far the true value might plausibly lie from it—a limitation that matters directly for a safety-oriented interpretation of temperatures “below an ignition threshold”. Third, the ten-highest-frame averaging convention used to summarize each run does not account for the fact that the number of recorded frames varies between runs, from 8 to 362. Consequently, nominally comparable summary statistics correspond to different, run-dependent fractions of the underlying temperature distribution.

More broadly, while artificial neural networks and other flexible regression models have been successfully applied to temperature prediction in machining, their applicability is less clear for datasets with a small sample size and one-factor-at-a-time structure characteristic of the present experiment, where only six to nine distinct technological levels are available for an individual parameter path. Increasing model flexibility under these conditions does not necessarily improve out-of-sample prediction and may instead increase the risk of overfitting. Therefore, model complexity should be evaluated on held-out observations rather than inferred from in-sample goodness-of-fit alone. The importance of model selection under limited experimental data availability has also been highlighted in other manufacturing applications, including friction drilling, where different machine-learning approaches were compared using a small experimental dataset [27].

This study extends the descriptive analysis of the experimental record by evaluating run-level predictive performance and predictive uncertainty. Its contribution comprises four elements: (1) the use of a consistently defined upper-tail response, T95, instead of a fixed number of the highest readings; (2) empirical evaluation of GP prediction intervals and a clipped jackknife+-type construction, with explicit recognition that the modified intervals do not retain the standard distribution-free coverage guarantee; (3) a leave-one-out comparison of linear, quadratic, Gaussian-process and neural-network regressors; and (4) an explicit distinction between predictions evaluated along the sampled single-parameter paths and an exploratory additive extension to untested combinations.

The aim of this study is to develop and evaluate a predictive model of the upper tail of chip temperature—expressed not only through point predictions but also through empirically evaluated prediction intervals—as a function of cutting speed, feed per tooth, and axial depth of cut for two rake angles (γ = 5° and γ = 30°) and two magnesium alloys (AZ31B and AZ91D), using the experimental dataset reported in [23]. Specifically, the study seeks to (1) construct a consistently defined run-level response despite differences in the number of recorded frames; (2) evaluate point-prediction errors and empirical prediction-interval coverage under leave-one-out validation; (3) compare simple and more flexible regression models at the sample size imposed by the experiment; and (4) illustrate the limitations of combining single-parameter models into a multi-parameter additive representation.

## 2. Materials and Methods

**Figure 1:**
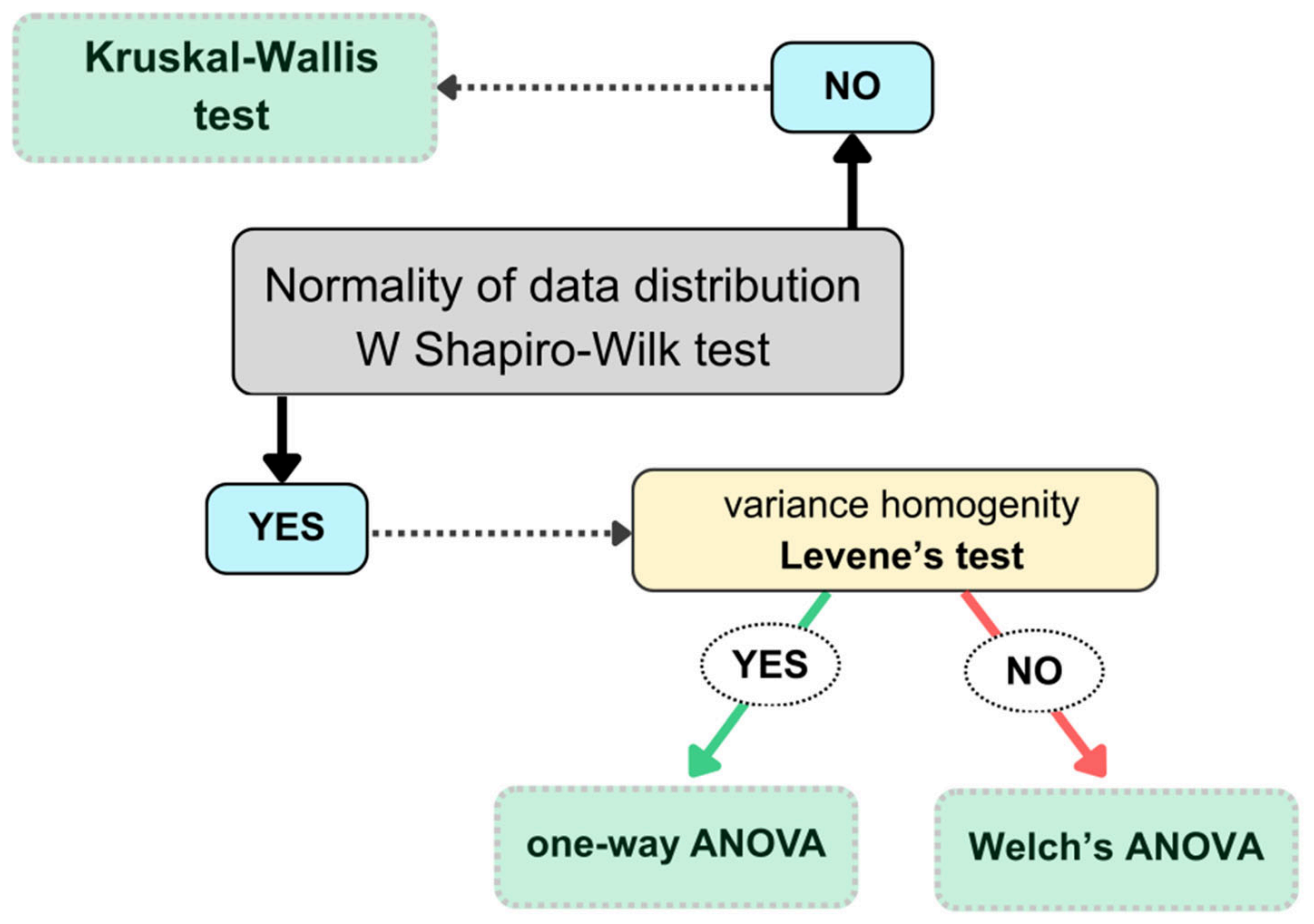
Diagnostic workflow used in the original frame-level analysis; the resulting statistics are retained as exploratory summaries only.

The machining run was the experimental unit. The original frame-level analysis used the ten highest available thermographic readings from each run, following the convention of [23]. Thus, six-level paths contributed 60 readings and nine-level paths contributed 90 readings, except for AZ31B/γ = 30°/v_c_, which contributed 88 because one run contained only eight valid frames. These readings are repeated measurements within a run, not independent experimental replicates. The original Shapiro–Wilk and Levene diagnostics and the corresponding ANOVA, Welch ANOVA and Kruskal–Wallis calculations are retained as exploratory summaries (Figure 1 and Table 1, Table 2, Table 3 and Table 4). They do not establish statistically significant run-level machining effects. Effect-size indices describe separation within the selected recordings and do not resolve within-run dependence or quantify between-run reproducibility. Most parameter levels have only one run record, limiting replicated inference across levels. Predictive modeling uses one T95 value per run.

For the classical one-way ANOVA, η^2^ was calculated as the ratio of the between-group sum of squares to the total sum of squares. For the Kruskal–Wallis summaries, the rank-based index η^2^_H_ was calculated as

(1) $${\eta^{2}}_{H}=\frac{H-k+1}{N-k}$$

where H is the Kruskal–Wallis statistic, k is the number of parameter levels, and N is the total number of selected temperature readings. Thus, k = 6 for the feed-per-tooth and axial-depth-of-cut paths and k = 9 for the cutting-speed paths. N denotes the number of selected readings, not the number of independent machining runs.

For the Welch summaries, an approximate F-based index was calculated as

(2) $$\omega^{2}=\frac{d_{f1}\cdot (F-1)}{d_{f1}\cdot F+d_{f2}+1}$$

using the reported Welch statistic and its numerator and denominator degrees of freedom. This quantity is reported solely as a descriptive approximation.

Table 1 documents the diagnostic route used in the original frame-level analysis. These diagnostics are retained for transparency and do not establish the validity of inferential testing for the dependent readings within each run. All statistical analyses were performed using Statistica software (Palo Alto, CA, USA), version 13.

The chip-surface temperature was recorded by infrared thermography (FLIR SC 6000 HS, Wilsonville, OR, USA, nominal rate 800 Hz) during dry milling with carbide end mills of two rake angles (γ = 5° and γ = 30°; helix angle 35°, clearance angle 8°, diameter 16 mm, z = 3). Three technological parameters were varied one at a time about a common central setting (cutting speed v_c_ = 800 m/min, feed per tooth f_z_ = 0.15 mm/tooth, axial depth-of-cut a_p_ = 6 mm; radial depth-of-cut a_e_ = 14 mm held constant throughout): v_c_ ∈ {400, 500, …, 1200} m/min (9 levels), f_z_ ∈ {0.05, 0.10, …, 0.30} mm/tooth (6 levels), and a_p_ ∈ {0.5, 1.0, 1.5, 3.0, 4.5, 6.0} mm (6 levels). For each alloy–rake-angle combination, the design comprised 21 run records at 19 unique technological settings, including three records at the shared central setting. Across the four alloy–rake-angle combinations, this gave 84 run records.

The camera was placed 2.14 m from the target and fitted with a 50 mm lens (F/2.3) for the 3–5 µm band. The acquisition protocol reported in [23] used a 128 × 160-pixel window and recordings of 800 frames at a nominal rate of 800 Hz (1 s). To evaluate the experimental results, a single temperature setting within the range of 150–350 °C was mainly used, with a short integration time and the dynamic range extension function enabled. In the Research IR 4.20.2.74 software (64-bit; Wilsonville, OR, USA), it was possible to analyze frames separately for both measurement ranges. In the event of a missing signal in a frame recorded within the 150–350 °C range, data from the lower setting was taken into account (10–90 °C). Another reason for using dynamic range extension was the ability to investigate the temperature of the workpiece during the milling tests on magnesium alloys. This measure was intended to facilitate the detection of the cutting tool and the workpiece within the machining zone, reduce testing costs and shorten the time required to carry out thermal imaging measurements. The authors also intended to use the data obtained to analyze the temperature distribution on the surface of the workpiece. These results are not the subject of this publication and may be used in future publications by the authors. The emissivity of each alloy was determined by calibration in a tube furnace (150–350 °C in 50 °C steps, at steady state) and set to ε = 0.31 for AZ31B and ε = 0.24 for AZ91D; it was treated as constant, because above 350 °C surface oxidation distorted the readings [23,28]. The temperature of each frame is the maximum within a 3 × 3-pixel analysis box placed on the chip-formation region just above the workpiece surface. The camera was focused on the front surface of the specimen, so that both the specimen and the chips passing through the focal zone were recorded. The number of chips in sharp focus in a given frame was random, and the recorded peak values also depended on the chip position and its distance from the focal plane. The highest available frame rate was therefore used to reduce the unfavorable effect of this variability and to capture high chip temperatures in as many frames as possible. The reported values are apparent chip-surface temperatures. The resolution of the thermal image was 160 × 128 pixels. For the 10–90 °C preset, the integration time was 0.3480 ms. For the 150 to 350 °C preset, the integration time was 0.009 ms. Two presets were configured with a dynamic range expansion function. Pixel size: 0.64 mm.

For each of the 84 runs, the frame-wise sequence of peak chip-temperature readings recorded during the corresponding machining pass was extracted from the original acquisition spreadsheets. Because acquisition and post-processing trimmed each recording to the interval containing genuine cutting engagement, the number of valid frames per run, N, is not fixed: across the 84 runs, N ranges from 8 to 362 (mean ≈ 79). Two datasets were derived from this frame-level record: a frame-level dataset retaining every valid reading together with its run’s technological parameters (6637 observations across the 84 runs), and a run-level dataset summarizing each run’s frames into the distributional statistics defined below (84 rows). As an internal consistency check, the maximum frame-level reading of each run was compared against the previously published peak chip-temperature values for the same conditions, the maximum discrepancy across all 84 runs was 0.005 C. The study [23] characterized each run by the mean of its ten highest recorded temperatures (“T_max, av_”). Because N varies between 8 and 362 across runs, a fixed count of ten highest readings corresponds to a different, run-dependent percentile of the underlying distribution and is therefore not directly comparable across runs. To address this, each run in Dataset B was instead characterized by empirical quantiles of its full frame-level distribution, computed via linear interpolation of order statistics (the standard ‘Type 7’ definition). A nominal 95% percentile-bootstrap interval for T95 was additionally computed for each run (2000 resamples with replacement, fixed random seed for reproducibility), providing an exploratory measure of sensitivity to resampling the observed frames. These intervals are exploratory because resampling individual frames ignores within-run temporal dependence. They do not include calibration uncertainty, spatial-resolution effects or between-run variability and should not be interpreted as complete 95% measurement-uncertainty intervals. Runs with N < 15 (2 of 84 runs) were flagged because their T95 estimates are close to the sample maximum and their confidence intervals are correspondingly wide (up to ≈37 °C in the most extreme case); results for these runs should be interpreted with caution. A sensitivity analysis excluding these two runs is reported in Section 3.

The experimental design varies exactly one technological parameter at a time about a shared central setting (a “star” design), meaning that no run exists in which two or more of v_c_, f_z_, and a_p_ simultaneously differ from their central value. This constraint directly shaped the modeling strategy: the primary analysis treats each of the three technological parameters, for each of the four alloy–rake-angle combinations, as an independent one-dimensional regression problem (12 curves in total: 3 parameters × 2 alloys × 2 rake angles), and predictions are restricted to the empirically tested range of the corresponding parameter. An additive combination of the twelve fitted curves into a single function of three variables was explored separately and is explicitly reported as an exploratory extension subject to the extrapolation risk inherent to the OFAT design.

For each of the 12 technological paths, Gaussian process (GP) regression was used to model T95 as a function of the varying parameter. A squared-exponential covariance function with an explicit linear basis function was used, and the hyperparameters (kernel length scale, signal variance, and observation noise variance) were estimated by maximum likelihood. Inputs were standardized prior to fitting. The models were fitted with the fitrgp function (MATLAB R2024b, Statistics and Machine Learning Toolbox) using a constant (homoscedastic) noise variance, exact fitting and prediction, the default initial values of the hyperparameters and the quasi-Newton optimizer. The hyperparameters were re-estimated from scratch within every cross-validation fold. The same kernel, basis function and optimizer settings were used for all twelve curves. The modelling analysis was retrospective. The hyperparameters estimated from the complete 6–9 point curves are reported in Section 3. GP regression was selected over more flexible regressors because it provides closed-form predictive uncertainty for each queried input, which is essential given that each curve comprises only 6 (f_z_, a_p_) or 9 (v_c_) distinct technological levels.

Given that each of the 12 curves is defined by only 6–9 distinct run records, leave-one-out cross-validation (LOO-CV) was used throughout, maximizing the information used for both fitting and validation at this sample size. For each curve, the run held out in a given fold contributes exactly one point to the pooled prediction-error metrics, computed as the mean absolute error (MAE), root-mean-square error (RMSE), coefficient of determination (R^2^, computed against the LOO predictions), mean relative error, and—for interval-producing models—the empirical coverage and mean width of the associated interval, and the maximum absolute error observed. The three runs at the shared central technological setting (v_c_ = 800 m/min, f_z_ = 0.15 mm/tooth, a_p_ = 6 mm) each appear once, in three different curves; since each curve is modeled and cross-validated independently, this does not introduce LOO-CV leakage. An initial evaluation of the naïve GP predictive intervals yielded a mean empirical coverage of 63.4% across the twelve curves, indicating under-coverage relative to the nominal 95% target. To assess an alternative without a held-out calibration set, a clipped modification of the jackknife+ procedure was applied [24], which combines the n leave-one-out models and their residuals to build a prediction interval whose theoretical, distribution-free coverage is at least 1–2α (90% for α = 0.05) when the data are exchangeable. Two qualifications apply at the present sample size. First, the textbook jackknife+ limits require the ⌈(1 − α)(n + 1)⌉-th smallest of the n shifted leave-one-out predictions, which exists only for n ≥ 19 when α = 0.05; for n = 6–9 (and n − 1 = 5–8 in the nested evaluation) the formal interval would be unbounded. Finite intervals were obtained by clipping the order-statistic index to the range 1…n, i.e., by using the smallest and largest shifted predictions, without interpolation. The 1–2α guarantee does not hold for these clipped intervals, and their coverage is assessed only empirically. Second, the guarantee relies on exchangeability, which cannot be justified for a fixed design in which every run occupies a different, deliberately chosen level of the parameter. The procedure is therefore used as an empirical calibration device and not as a source of a coverage guarantee. Coverage was evaluated by nested double LOO: the outer test run was excluded from fitting and from the inner residual pool. Inner fits use n − 2 runs and yield n − 1 interval candidates. The reported median inner-model point predictor differs from the ordinary GP predictor fitted on n − 1 runs. Endpoint folds evaluate extrapolation beyond the training range, whereas interior folds evaluate interpolation. To provide a practical (though explicitly non-validated) means of estimating T95 for arbitrary combinations of v_c_, f_z_, and a_p_—including combinations not directly tested—the twelve GP curves were combined, per alloy–rake-angle group, into an additive function

(3) $$T(v_{c},\;f_{z},\;a_{p})=G_{v}(v_{c})+G_{f}(f_{z})+G_{a}(a_{p})-2\beta_{0}$$

where G_v_, G_f_, G_a_ are the fitted GP curves for that group and $\beta_{0}$ is the mean of the three curves’ own GP-smoothed predictions at the shared central setting. This exploratory construction is motivated by the star-design geometry: because each path holds the other two parameters at their central value, each measures T = β0 + g_i_(parameter) directly, and summing three such measurements counts β0 three times, which Equation (3) corrects. Here, β0 is chosen from the smoothed central predictions; this is a modeling choice. When those predictions disagree, the additive surface cannot exactly reproduce all three component curves along their axes. The equation assumes the complete absence of interaction effects between v_c_, f_z_, and a_p_—an assumption the OFAT design can neither support nor refute. Predictions for combinations in which two or more parameters simultaneously depart from the central setting were accordingly flagged as extrapolative and are reported separately from (and are not to be conflated with) the validated single-parameter results. As a further diagnostic, the spread among the three curves’ own smoothed predictions at the shared central point was computed per group (range 4.8–59.6 °C across the four groups); a larger spread indicates a less internally consistent—and correspondingly less trustworthy—additive combination for that group, independently of the extrapolation concern.

To assess whether the choice of GP regression materially affects the conclusions, each of the 12 curves was additionally fitted, under the identical LOO-CV protocol, with ordinary least-squares linear regression, second-degree polynomial (quadratic) regression—the functional form used for trend approximation—and a shallow feed-forward neural network (one hidden layer, three neurons, hyperbolic-tangent activation, ridge penalty λ = 0.1). For the linear and quadratic models, prediction intervals were obtained in two ways: with the same clipped jackknife+-type procedure (identical clipping rule and nested evaluation) and as classical least-squares t-intervals, which assume homoscedastic Gaussian errors and were evaluated under the same leave-one-out protocol. The neural network was included specifically as a sensitivity/robustness check rather than a candidate model: with only 5–8 training points per LOO fold, a network of this size (10 free parameters) is nominally over-parameterized.

## 3. Results

Table 2, Table 3 and Table 4 summarize numerical differences between the selected upper-temperature readings across parameter levels within each alloy–rake-angle group. Because readings within a machining run are dependent, these calculations are interpreted descriptively and do not establish statistically significant run-level effects. They also do not constitute a direct statistical comparison between the 5° and 30° rake angles.

Inferential *p*-values and significance conclusions are withheld because readings within a machining run are not independent. When analyzing the values of the effect size indices η^2^ and ω^2^, which are used to assess the scale effect, the values of these indices for all analyses performed are greater than 0.14, indicating a large scale effect. When considering the influence of technological parameters, it should be noted that for both the AZ31 and AZ91 alloys, at both rake angles γ = 5° and γ = 30°, cutting speed ranks last in terms of its contribution to the effect size. The analysis showed that cutting speed has the least effect on cutting temperature; the result is determined by the feed rate f_z_ and the cutting depth a_p_. The feed rate f_z_ and the depth of cut a_p_ affect the cross-sectional area of the cutting layer and, consequently, the load on the tool edges, which is most likely to influence the amount of heat generated in the cutting zone and, consequently, the cutting temperature. It should also be noted that for the AZ91 alloy and a rake angle γ = 30°, all scale effect indices are high (above 0.70).

The descriptive statistical analysis and predictive modeling addressed complementary questions. ANOVA/Welch ANOVA/Kruskal–Wallis tests were retained as numerical descriptions of differences in recorded chip temperatures across the applied technological settings, whereas the subsequent modeling stage focused on the prediction of the run-level upper-tail statistic T95 and its uncertainty. Because the frame-level tests do not account for the grouping of readings within runs, they were not used to support the modelling conclusions; the predictive part of the study relies exclusively on run-level data (one value per run).

Under leave-one-out cross-validation, the nominal 95% predictive interval produced directly by the fitted GP posterior achieved an empirical coverage of only 63.4%, averaged over the 12 technological paths (range 33–89% across individual curves; Table 5). The coverage fell to 33% for the AZ91D/γ = 30°/a_p_ curve and to 50% for four other curves, showing under-coverage in this evaluation without separately identifying its cause. This finding motivated the conformal calibration described in Section 2.

**Figure 2:**
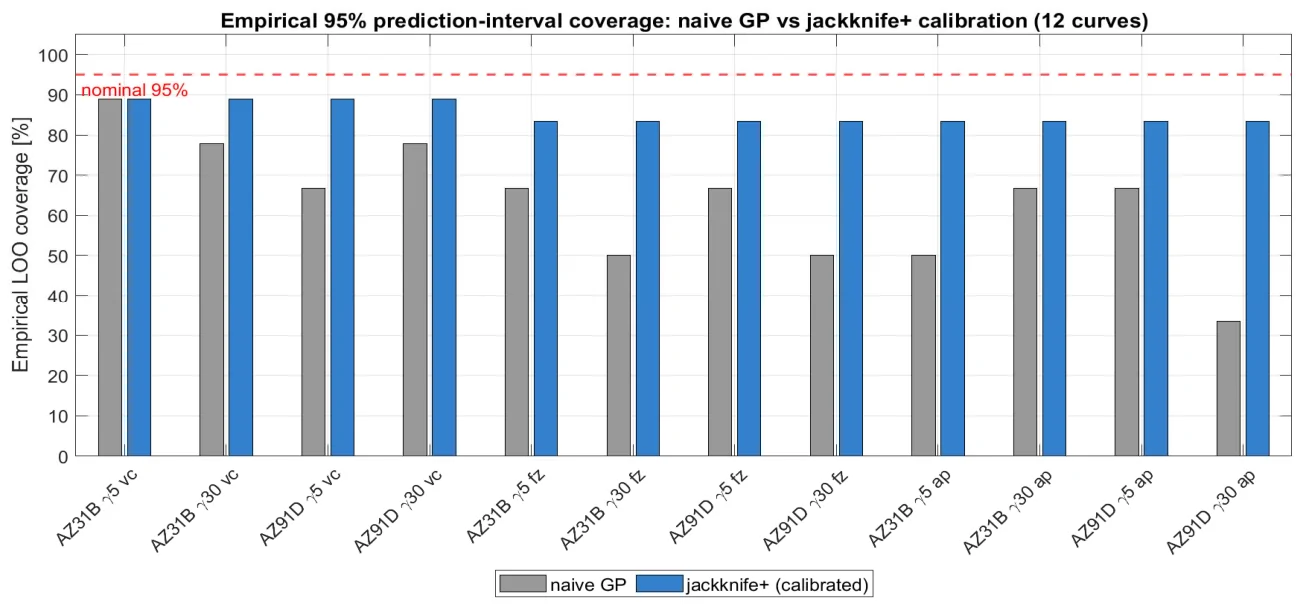
Empirical leave-one-out coverage of the nominal 95% prediction interval for each of the twelve technological paths, before (naive GP) and after clipped jackknife+-type procedure, against the nominal 95% target (dashed line).

The observed nested-LOO coverage reached 83.3% for six-level curves and 88.9% for nine-level curves. Three levels of coverage should be distinguished: the nominal target of 95%; the 90% (1–2α) bound of the jackknife+ theory, which does not formally apply here because clipped intervals are used at n < 19 and the exchangeability of runs in a fixed OFAT design cannot be justified; and the empirically observed 85.2% (mean over the curves; 72 of 84 held-out runs, i.e., 85.7% folds). The calibrated intervals are thus much better calibrated than the raw GP intervals, but they should not be regarded as reliable 95% prediction intervals. Because coverage can change only in increments of 1/6 or 1/9 at these sample sizes, the estimates for individual curves are, in addition, coarse. The correction was not without cost: mean interval width nearly doubled, from 38.4 °C (naïve GP) to 73.0 °C (JK+); point-prediction accuracy changed only marginally (mean MAE 15.4 → 14.7 °C; mean RMSE 18.4 → 17.6 °C), because the reported point predictor also changes to the median of inner-model predictions (Section 2); interval calibration alone does not change point predictions (Table 5, Figure 2). Excluding the two runs flagged with N < 15 did not change these conclusions: the naïve coverage was 62.6% (mean width 38.6 °C), the jackknife+ coverage 85.8% (76.1 °C), and the mean MAE 15.88 °C for GP and 15.18 °C after jackknife+. The sampling uncertainty of the response itself is not negligible: the bootstrap 95% interval of T95 had a median width of 17.9 °C (90th percentile 35.9 °C, maximum 53.7 °C), which is of the same order as the mean leave-one-out error (about 15 °C), so response-estimation uncertainty may contribute to prediction error, but its contribution is not separated from model error.

**Figure 3:**
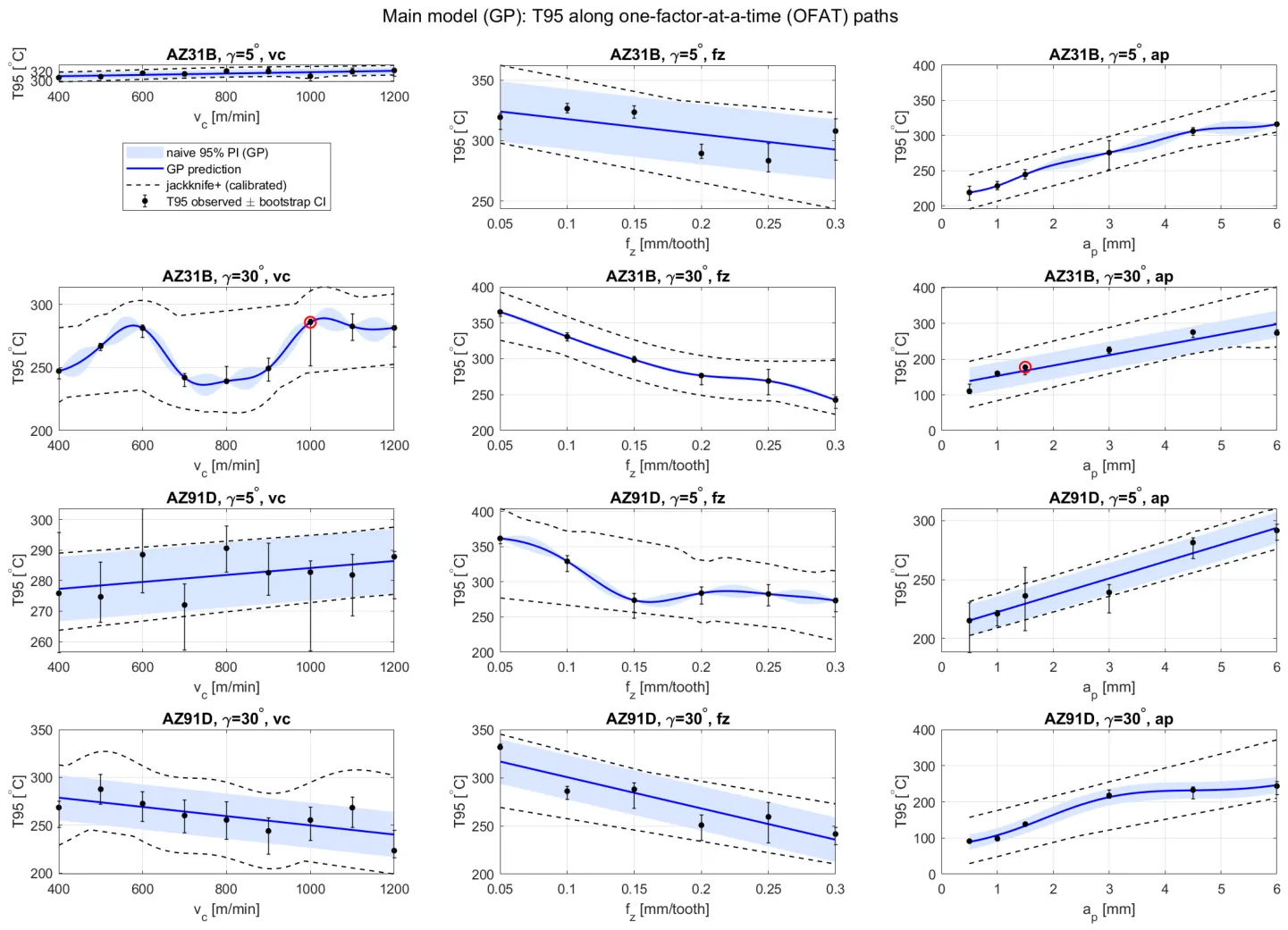
GP regression fits for the twelve one-factor-at-a-time technological paths (rows: alloy/rake-angle group; columns: v_c_, f_z_, a_p_).

Figure 3 shows the twelve fitted curves together with the raw naïve GP band (light shading), the clipped jackknife+-type band (dashed lines), and the experimental T95 values with their exploratory frame-resampling intervals; the visual gap between the two bands directly illustrates the magnitude of the calibration correction reported above. Red circles: runs with N < 15.

**Figure 4:**
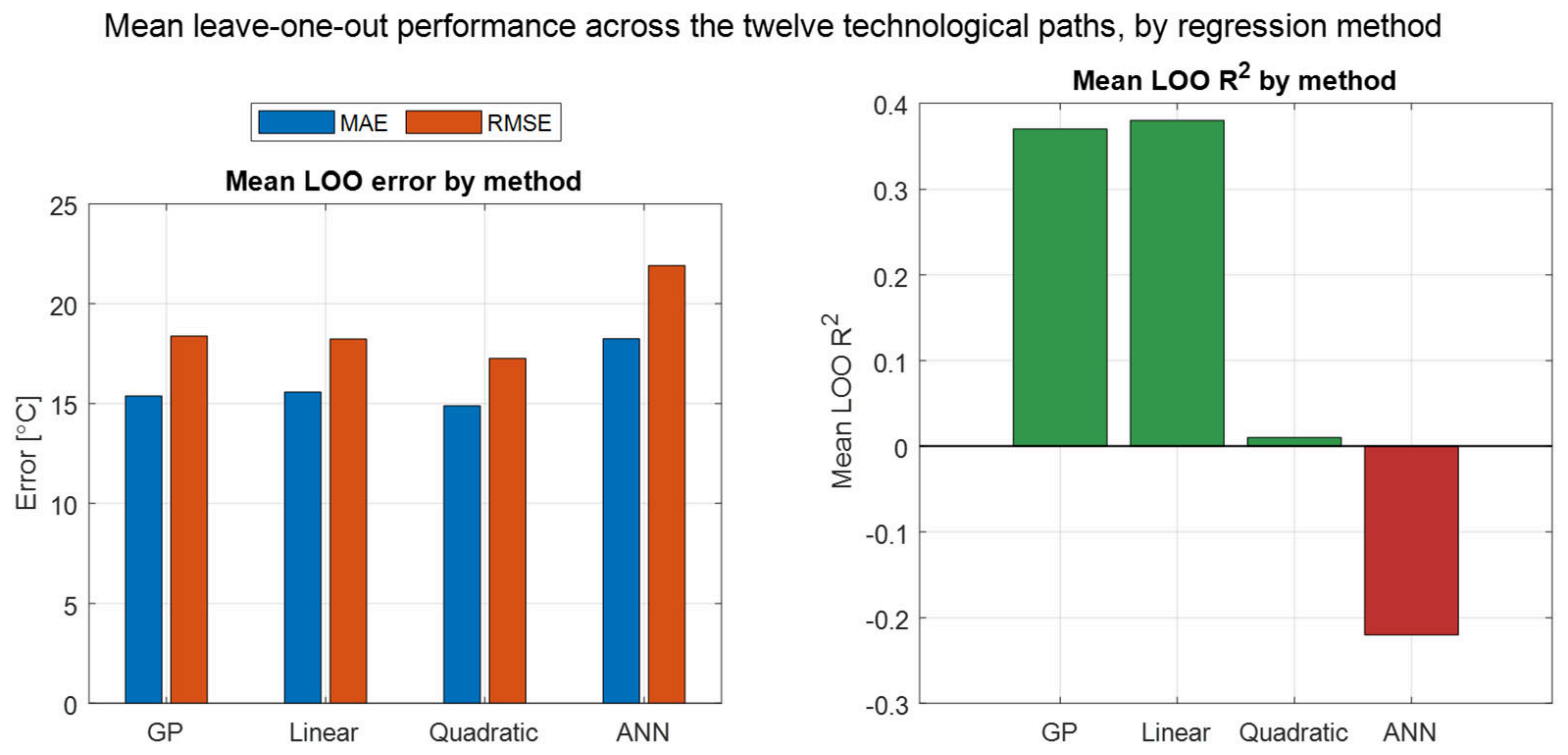
Mean leave-one-out MAE, RMSE, and R2 across the twelve technological paths for each of the four regression methods compared in this study.

Linear and quadratic regression, fitted under the same LOO-CV protocol, produced comparable mean point-prediction accuracy from GP (mean MAE 15.6 °C and 14.9 °C, respectively, versus 15.4 °C for GP; Table 6, Figure 4) but, like GP, can be equipped with prediction intervals (Table 7). The shallow neural-network sensitivity check underperformed all three other methods (mean MAE 18.2 °C, mean R^2^ = −0.22) and improved on GP in only 2 of the 12 curves, confirming that increased model flexibility is not beneficial at the present sample size. Although quadratic regression achieved the lowest mean MAE (14.89 °C), its substantially lower mean LOO R^2^ (0.01) was largely driven by the AZ31B/γ = 5°/fz curve (R^2^ = −3.81), where a single held-out endpoint prediction showed an error of 61 °C (Table 8). Since R^2^ normalizes squared prediction errors by the observed response variability within each curve, this poor curve-level result strongly reduced the mean R^2^ despite the slightly lower average absolute prediction error.

To compare the uncertainty quantification of the models on an equal footing, clipped jackknife+-type intervals (same clipping rule and nested evaluation) and classical least-squares prediction intervals were also computed for the linear and quadratic models (Table 7). With jackknife+, linear regression reached the same mean coverage as GP (85.2%) at practically the same mean width (73.1 vs. 73.0 °C), whereas the quadratic model reached 86.6% at a considerably larger width (104.1 °C). The classical t-intervals of the linear and quadratic models attained 95.4% and 94.0% coverage, respectively, close to the nominal level, but were wider (mean 101.3 and 123.9 °C). The ability to produce a calibrated interval is therefore not specific to Gaussian process regression. At n = 6–9, the GP is in effect a linear-basis regression with a very small kernel contribution (signal standard deviation σf < 1 °C in 7 of the 12 curves, Table 9), which is consistent with its point predictions and clipped jackknife+-type intervals being almost indistinguishable from those of linear regression.

Mean MAE and mean R^2^ ranked the methods differently (Table 6) because the two metrics aggregate differently across the curves. The curve-level results (Table 8) show that the mean R^2^ of the quadratic model (0.01) is dominated by a single curve, AZ31B/γ = 5°/f_z_ (R^2^ = −3.81), where one held-out run (f_z_ = 0.30 mm/tooth, at the edge of the tested range, error +61 °C) accounts for 46% of the sum of squared errors; without this curve the mean R^2^ is 0.35 for the quadratic model, 0.44 for GP and 0.45 for linear regression, and the median R^2^ over all twelve curves is 0.58, 0.45 and 0.45, respectively. The differences between the methods are not uniform: the quadratic model had the lowest MAE in 5 of the 12 curves, all of them f_z_ or a_p_ paths (mean MAE for a_p_: 13.0 vs. 18.7 °C for GP), but it was worse on the v_c_ paths (12.3 vs. 9.7 °C). For the quadratic model, the largest-error fold was an end point of the tested range in 10 of the 12 curves, i.e., the leave-one-out error is dominated by extrapolation to the edge of the range.

The hyperparameters of the Gaussian process models fitted to the complete 6–9 point curves are listed in Table 9 (the same settings were re-estimated within each cross-validation fold). The length scale ℓ is expressed in units of the standardized input; σf (signal standard deviation), σn (noise standard deviation) and the linear-basis coefficients are in °C (β1 per standard deviation of the input). Values of σf below 1 °C (7 of 12 curves) mean that the model has collapsed to a linear-basis regression with homoscedastic noise. The length scales range over two orders of magnitude (0.05–8.32), this variation alone does not establish identifiability at n = 6–9.

Aggregating Table 5 by technological parameter shows that the cutting-speed paths had the lowest absolute prediction errors and the smallest mean interval widths. Mean GP MAE was 9.7 °C for v_c_, versus 17.7 °C for f_z_ and 18.7 °C for a_p_. The gap widened further after calibration: mean jackknife+ interval width was 43.8 °C for v_c_, compared with 80.6 °C for f_z_ and 94.5 °C for a_p_—the a_p_ paths requiring, on average, intervals more than twice as wide as the v_c_ paths to achieve comparable coverage. The largest curve-level mean interval width (161.5 °C) was observed for the AZ91D/γ = 30°/a_p_ path. This asymmetry—rather than any single point estimate—is the clearest quantitative signature in the present data of parameter-dependent uncertainty in the upper tail of the chip-temperature distribution.

Repeating the full pipeline with T_max, av_ as the response variable in place of T95 produced closely comparable results, with both the naïve-coverage deficit and its correction by jackknife+ calibration converging to the same 85.2% mean coverage—showing that the pattern is not confined to T95, without establishing its cause.

The additive combination retained the shapes of the component curves but introduced path-dependent offsets due to disagreement among their predictions at the shared central setting. This disagreement was small for AZ31B/γ = 5° (4.8 °C) and AZ91D/γ = 5° (19.9 °C), but substantial for AZ91D/γ = 30° (38.0 °C) and especially AZ31B/γ = 30° (59.6 °C)—the latter driven by the three raw replicate observations of the nominal central condition themselves spanning 239–299 °C. Illustrative bands without an established coverage guarantee for these predictions, obtained by summing the half-widths of the three constituent jackknife+ intervals, ranged from ± 66 °C to ± 175 °C.

**Figure 5:**
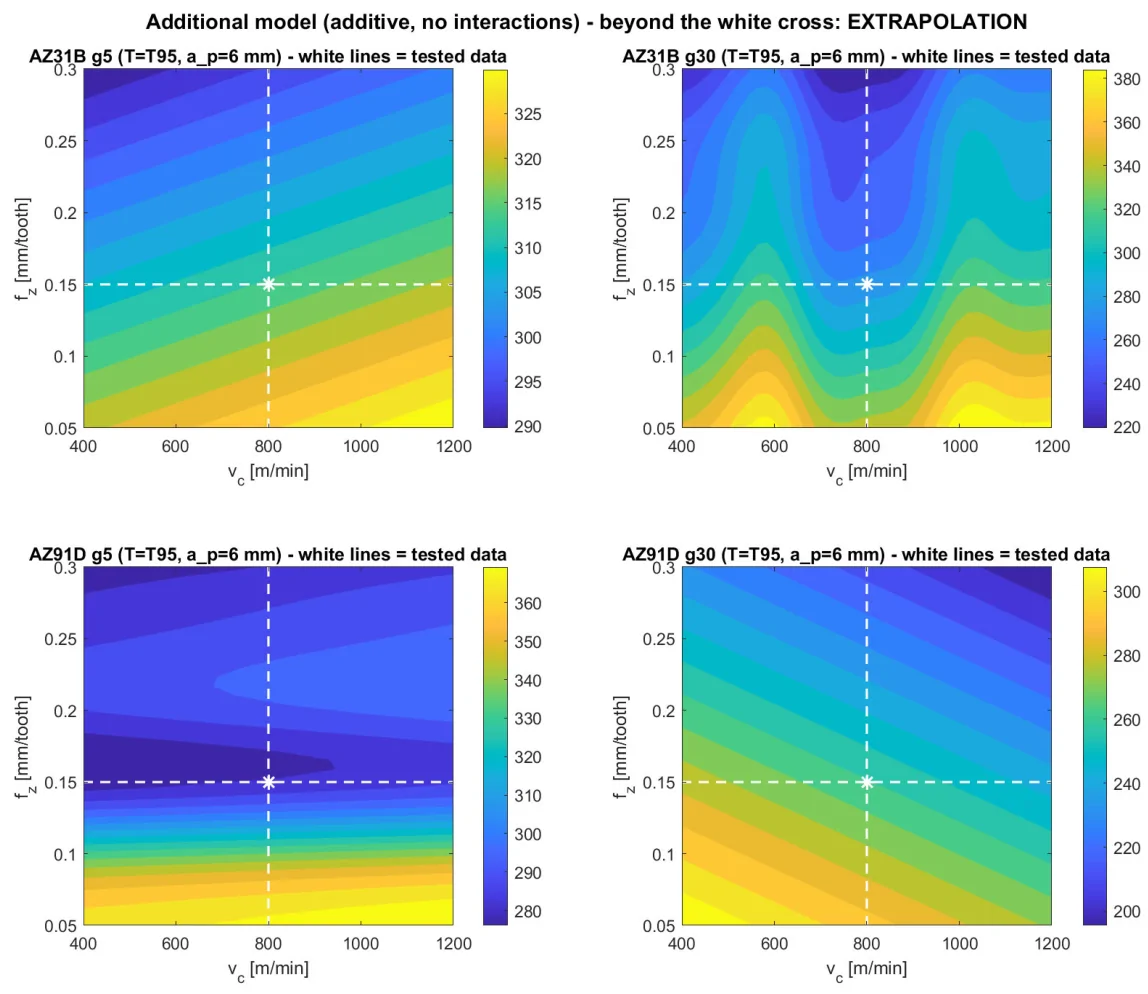
Exploratory additive-model response surfaces T(v_c_, f_z_) at a_p_ = 6 mm, one panel per alloy–rake-angle group.

Figure 5 presents the resulting T(v_c_, f_z_) response surfaces at a_p_ = 6 mm for the four alloy–rake-angle groups, with the tested “cross” of OFAT data explicitly overlaid to distinguish the experimentally sampled paths from the surrounding, purely extrapolative surface. For AZ31B/γ = 30°, the surface reproduces the non-monotonic, three-subrange behavior along v_c_ already noted qualitatively in [23].

## 4. Discussion

The naïve GP posterior intervals achieved a mean empirical coverage of only 63.4% across the twelve curves, despite a nominal target of 95%. Had the study only involved fitting a GP and reporting its native interval, as is common practice in the small-sample machining-temperature literature, the resulting ‘95% confidence’ statements would have been misleading by a wide margin. The clipped procedure approximately doubled interval width and increased observed coverage; this does not quantify the true uncertainty or establish 95% coverage. This difference is important for interpreting the data in a safety-oriented way. Rather than any specific point-prediction metric, this is regarded as the principal contribution that distinguishes the present model-based study from the purely descriptive analysis of [23]. At the same time, the calibrated intervals reached only 85.2% coverage, which is below the nominal 95% and below the 90% bound of the jackknife+ theory (which does not formally apply at n = 6–9 for a fixed design). They should be read as an improved but still approximate indication of uncertainty, not as reliable 95% prediction intervals.

Within the sampled ranges, the feed-per-tooth and axial-depth-of-cut paths had higher absolute prediction errors and wider intervals than the cutting-speed paths. Mean GP MAE was 9.7 °C for v_c_, compared with 17.7–18.7 °C for f_z_ and a_p_; the corresponding mean interval widths were 43.8 °C and 80.6–94.5 °C. These comparisons describe prediction errors and interval widths in temperature units. Differences in response variation, the number of sampled levels and run-to-run variability may contribute to this pattern; the present analysis does not isolate its physical cause. Rake angle did not uniformly increase or decrease predictability; instead, it modified the relationship in a parameter-specific direction. For v_c_ and a_p_, the γ = 30° curves were consistently harder to model than their γ = 5° counterparts (e.g., v_c_: MAE 3.9–5.2 °C at γ = 5° versus 14.4–15.4 °C at γ = 30°); for f_z_, the opposite held (γ = 30° MAE 13.1–16.7 °C versus γ = 5° 18.4–22.7 °C). This asymmetry is consistent with the chip-geometry mechanism proposed in [23], thin, reactive chips at γ = 30° versus a chip-swelling effect at γ = 5°, acting differently on the sensitivity of the upper temperature tail to speed/depth-of-cut than to feed.

The two alloys likewise were modelled with distinct predictive functions; a shared model was not evaluated. Beyond the qualitatively different v_c_ trends already reported in [23] (a non-monotonic, three-subrange response for AZ31B at γ = 30° versus a smoother trend for AZ91D under the same conditions, reproduced here in Figure 3 and in the corresponding panel of Figure 5), the two alloys’ fitted baseline levels differed by 30–35 °C within each rake-angle group, and their error/coverage profiles across the twelve curves did not track one another.

By contrast, moving to a more flexible nonlinear model did not improve predictive accuracy over simpler regression approaches, while the companion study [23] used trend-line models for descriptive approximation of the experimental relationships. To the authors’ knowledge, this is a genuinely negative result that is worth stating plainly: under rigorous leave-one-out validation, neither GP regression nor quadratic regression achieved a meaningfully lower mean absolute error (MAE) than ordinary linear regression (15.4 °C, 14.9 °C, and 15.6 °C, respectively; see Table 6). Furthermore, the mean R^2^ of the quadratic model (0.01) was markedly worse than either alternative, although this mean is dominated by one curve with a single influential fold (AZ31B/γ = 5°/f_z_, Table 8), and the quadratic model had the lowest MAE in 5 of the 12 curves, mostly a_p_ paths. The extra curvature parameter thus helps on some paths and hurts on others, rather than being uniformly beneficial at n = 6–9. Combined with the shallow-network sensitivity check, which performed worse than all three simpler methods, this indicates that the benefit of moving beyond [23]’s second-degree polynomial trend lines lies not in superior point-prediction accuracy—none of the methods tested here reliably deliver that at the present sample size—but in the explicit calibration of predictive uncertainty. This benefit is not specific to the Gaussian process: jackknife+ can be applied to any of the regressors, and classical least-squares prediction intervals are available for the linear and quadratic models (Table 7). GP regression was retained as the primary model because its posterior provides a native, input-dependent uncertainty estimate and contains the linear model as a limiting case. With a signal standard deviation below 1 °C in 7 of 12 curves (Table 9), it effectively reduces to that case, and the present data do not demonstrate an advantage of the GP over linear regression.

Uncertainty assessment was limited both by the number of run-level observations per curve and by the number of frames available within each run. This is corroborated by three observations in this study: (i) the parameter-dependent gap in interval width described above; (ii) the coarse resolution of observed coverage (8/9 = 88.9% on nine-level curves and 5/6 = 83.3% on six-level curves; coverage can change only in increments of 1/9 or 1/6, but these fractions are not an upper limit); and (iii) the two explicitly flagged low-N runs (N = 8 and N = 11), whose frame-level bootstrap confidence intervals for T95 were among the widest observed (up to ≈ 37 °C from resampling uncertainty alone, before any curve-fitting uncertainty is even considered).

Prior applications of artificial neural networks to temperature prediction in machining (e.g., turning of Al-7075, [29]) have reported favorable results relative to simpler regressors, typically on datasets with substantially more training observations than are available here; the present finding that a shallow ANN underperforms linear regression applies to the tested network and dataset, rather than contradicting that literature. At n = 6–9 distinct technological levels, the tested increase in model flexibility did not consistently improve prediction, reinforcing the view that method selection here should be driven by the design’s size and structure rather than by a presumption that more flexible learners are categorically preferable.

For the tested paths, cutting speed had smaller curve-level mean interval widths (20–71 °C) than feed per tooth or axial depth of cut (up to 162 °C). These empirical differences in predictability do not establish safety margins or justify quantitative process-optimization recommendations. The exploratory additive surfaces may assist qualitative screening of untested parameter combinations but must not be used for quantitative safety decisions, given their unvalidated no-interaction assumption and the substantial center-point inconsistency observed for two of the four alloy–angle groups (up to 59.6 °C). Likewise, none of the results presented here should be taken as evidence that the ignition risk is negligible below any given temperature.

Three limitations bound the scope of the conclusions above. First, the OFAT experimental design fundamentally precludes estimation or validation of interactions between v_c_, f_z_, and a_p_; every predictive claim in this study is confined to single-parameter variation about the tested central setting, and the additive extension is explicitly exploratory rather than validated. Second, coverage estimates are coarse at n = 6–9 and the clipped intervals have no standard coverage guarantee; additional independent runs are needed for a stronger evaluation. Third, the pronounced center-point inconsistency observed for the AZ31B/γ = 30° and AZ91D/γ = 30° groups indicates that run-to-run variability at fixed nominal settings is itself non-negligible for these conditions, a source of uncertainty that is not captured by the frame-level bootstrap interval on T95 and is not separately decomposed from model uncertainty in the present analysis.

The conclusions are also limited by the measurement. The analysed temperatures are apparent chip-surface temperatures measured by infrared thermography on small chips moving at high speed. Their uncertainty depends on the emissivity, which was determined for each alloy between 150 and 350 °C and treated as constant (above 350 °C surface oxidation distorted the calibration readings), on the spatial resolution relative to the chip size (the analysis box was 3 × 3 pixels) and on the integration time and frame rate relative to the chip transit time (both of which tend to lower the apparent peak through averaging), and on the random number of chips in the focal zone. T95 should therefore be read as an indicator of the upper tail of the recorded readings and not as the true maximum chip temperature. In addition, the camera preset covered 150–350 °C and was not calibrated beyond it, whereas 60 of the 6637 readings (in three runs, all at f_z_ = 0.05 mm/tooth) exceed 350 °C. Temperature values below 150 °C, as well as those above 350 °C, fall outside the nominal chip-measurement preset and the range used for emissivity calibration; their measurement uncertainty was not quantified in this reanalysis. In two of these runs the highest readings coincide at 365.2–365.3 °C, and in one of them (AZ31B, γ = 30°, f_z_ = 0.05 mm/tooth) 22 frames have an identical value of 365.2 °C. This pattern may reflect clipping or limited output precision, but detector saturation cannot be confirmed from exported readings alone; if clipping occurred, the top of the distribution is truncated and the temperatures of these hottest conditions are underestimated, and T95 and the ten highest readings of these runs are less reliable than those of the other runs. A chip-surface temperature below a cited ignition threshold does not demonstrate that ignition risk is negligible, because ignition depends also on the chip size, surface oxidation and thermal history. Finally, the tests of Section 3 use the ten highest readings per run, whereas the modelling uses T95; the two summaries differ, and the frame-level tests cannot account for run-to-run variability.

## 5. Conclusions

This study involved the development and validation of a predictive framework for the upper tail of chip temperature during the dry rough milling of AZ31B and AZ91D magnesium alloys, using tools with rake angles of 5° and 30°. The 95th percentile of the frame-level thermographic observations (T95) was adopted as the modelling target, providing a consistent upper-tail statistic across runs of different lengths. The selected upper-temperature readings differed across parameter levels within the alloy–rake-angle groups. The descriptive frame-level indices do not establish independently replicated machining effects.

The predictive analysis showed that increased model complexity did not systematically improve the accuracy of point predictions for the small experimental dataset available. Under leave-one-out cross-validation, Gaussian process regression achieved a mean MAE of 15.38 °C, compared to 15.57 °C for linear regression and 14.89 °C for quadratic regression. Meanwhile, the shallow neural network performed worse with a mean MAE of 18.24 °C. Linear and quadratic regression could be equipped with prediction intervals as well (jackknife+ coverage 85.2% and 86.6%; classical least-squares intervals 95.4% and 94.0% at larger widths). The cutting-speed paths had the lowest mean GP MAE (9.7 °C), compared with 17.7 °C for feed per tooth and 18.7 °C for axial depth of cut.

The nominal 95% intervals obtained directly from Gaussian process regression achieved a mean empirical coverage of only 63.4% across the twelve curves, showing substantial under-coverage in this evaluation. Although the clipped jackknife+-type procedure increased the mean empirical coverage to 85.2%, it also increased the mean prediction-interval width from 38.4 °C to 73.0 °C. This coverage remains below the nominal 95%, and the formal jackknife+ guarantee does not apply to this clipped procedure and exchangeability is not established by the fixed experimental design, so the calibrated intervals should be treated as approximate. These results suggest that evaluation of point-prediction accuracy alone is insufficient for small machining datasets and should be complemented by an explicit assessment and calibration of predictive uncertainty.

The results also demonstrate that the scope of valid prediction is determined by the experimental design. A one-factor-at-a-time design only permits predictive evaluation along the single-parameter paths that have been investigated experimentally. Therefore, the proposed additive multi-parameter model should be treated as exploratory, as interactions between cutting speed, feed per tooth and axial depth of cut cannot be identified from the present dataset. Accordingly, response surfaces generated outside the experimentally sampled paths should not be interpreted as validated quantitative predictions.

## Figures and Tables

**Table 1:** Types of tests used in the statistical analysis of the dependent variable depending on the milling conditions.

Material Grade	Dependent Variable as a Function of the Qualitative Factor	Test Type	Original Diagnostic Criterion
AZ31B	v_c_ for γ = 5°	Welch ANOVA	variances are heterogeneous
	f_z_ for γ = 5°	Kruskal–Wallis	for f_z_ = 0.05 mm lack of normality of distribution W = 0.60863; and for f_z_ = 0.10 W = 0.77847
	a_p_ for γ = 5°	Welch ANOVA	variances are heterogeneous
	v_c_ for γ = 30°	Kruskal–Wallis	for v_c_ = 800 m/min lack of normality of distribution W = 0.74048
	f_z_ for γ = 30°	Kruskal–Wallis	for f_z_ = 0.05 mm lack of normality of distribution W = 0.36572
	a_p_ for γ = 30°	Kruskal–Wallis	for a_p_ = 0.5 mm lack of normality of distribution W = 0.80871
AZ91D	v_c_ for γ = 5°	Kruskal–Wallis	for v_c_ = 700 m/min lack of normality of distribution W = 0.84342
	f_z_ for γ = 5°	Welch ANOVA	variances are heterogeneous
	a_p_ for γ = 5°	Welch ANOVA	variances are heterogeneous
	v_c_ for γ = 30°	one-way ANOVA	-
	f_z_ for γ = 30°	Welch ANOVA	variances are heterogeneous
	a_p_ for γ = 30°	Kruskal–Wallis	for a_p_ = 1.0 mm lack of normality of distribution W = 0.57483 and for a_p_ = 1.5 mm W = 0.64025

**Table 2:** Results of one-way ANOVA for the AZ91D alloy regarding the effect of cutting speed (v_c_) on cutting temperature, where df—number of the degrees of freedom; SS—sum of squares between groups; MS—mean sum of squares between groups; F—value of the test statistic.

AZ91 for γ = 30°					
Variable	df	SS	MS	F	Effect Size η^2^
v_c_	8	39,950	4994	29.2100	0.7426

**Table 3:** Results of Welch’s ANOVA regarding the effect of cutting speed (v_c_), depth of cut (a_p_), and feed per tooth (f_z_) on cutting temperature, where df1—degrees of freedom for the variance between groups; df2—degrees of freedom for the variance within groups; F—value of the test statistic.

Grade	γ [°]	Variable	df1	df2	F Welch	Effect Size ω^2^
AZ31	5	v_c_	8	33.47656	14.75684	0.7215
		a_p_	5	23.24164	827.7981	0.9930
AZ91	5	f_z_	5	21.66351	469.3676	0.9883
		a_p_	5	24.66684	162.3162	0.9634
	30	f_z_	5	24.36395	321.1848	0.9814

**Table 4:** Kruskal–Wallis test for the temperature in the cutting zone.

Grade	γ [°]	Variable		Effect Size η^2^_H_
AZ31	5	f_z_	KW H (5; N = 60) = 48.04459	0.7971
	30	v_c_	KW H (8; N = 88) = 63.16061	0.6982
		f_z_	KW H (5; N = 60) = 54.93091	0.9246
		a_p_	KW H (5; N = 60) = 54.21941	0.9115
AZ91	5	v_c_	KW H (8; N = 90) = 28.06330	0.2477
	30	a_p_	KW H (5; N = 60) = 53.90938	0.9057

**Table 5:** Leave-one-out cross-validation results for the twelve GP-modelled technological paths (target variable: T95).

Alloy	γ (°)	Parameter	n	GP MAE (°C)	Naïve Cov. (%)	Naïve PI Width (°C)	JK+ Cov. (%)	JK+ PI Width (°C)
AZ31B	5	v_c_	9	3.92	89	15.1	89	19.7
AZ31B	30	v_c_	9	14.38	78	52.2	89	60.7
AZ91D	5	v_c_	9	5.23	67	20.9	89	24.0
AZ91D	30	v_c_	9	15.38	78	45.4	89	70.8
AZ31B	5	f_z_	6	18.38	67	46.8	83	70.7
AZ31B	30	f_z_	6	13.13	50	17.1	83	53.0
AZ91D	5	f_z_	6	22.71	67	57.3	83	126.3
AZ91D	30	f_z_	6	16.72	50	43.2	83	72.4
AZ31B	5	a_p_	6	9.90	50	18.8	83	43.8
AZ31B	30	a_p_	6	29.10	67	63.5	83	129.5
AZ91D	5	a_p_	6	7.29	67	25.3	83	43.1
AZ91D	30	a_p_	6	28.47	33	55.6	83	161.5
Mean				15.38	63.4	38.4	85.2	73.0

**Table 6:** Mean leave-one-out metrics across the twelve technological paths, by regression method.

Model	Mean MAE (°C)	Mean RMSE (°C)	Mean R^2^
Gaussian process	15.38	18.38	0.37
Linear regression	15.57	18.23	0.38
Quadratic regression	14.89	17.26	0.01
Shallow ANN (sensitivity check)	18.24	21.91	−0.22

**Table 7:** Mean leave-one-out coverage and width of the nominal 95% prediction intervals over the twelve curves, by model and interval method (clipped jackknife+-type intervals evaluated with the nested leave-one-out procedure).

Model	Interval Method	Mean Coverage (%)	Mean Width (°C)
Gaussian process	Naïve posterior interval	63.4	38.4
Gaussian process	clipped jackknife+ type	85.2	73.0
Linear regression	clipped jackknife+ type	85.2	73.1
Linear regression	Least-squares t-interval	95.4	101.3
Quadratic regression	clipped jackknife+ type	86.6	104.1
Quadratic regression	Least-squares t-interval	94.0	123.9

**Table 8:** Curve-level leave-one-out MAE and R^2^ of the Gaussian process, linear and quadratic models, and the most influential fold of the quadratic model.

Alloy	γ (°)	Parameter	GP MAE (°C)	GP R^2^	Linear MAE (°C)	Linear R^2^	Quadratic MAE (°C)	Quadratic R^2^	Largest Quad. Error: Level (°C)
AZ31B	5	v_c_	3.92	0.19	3.90	0.19	4.21	−0.02	1000 (−11)
AZ31B	30	v_c_	14.38	0.12	17.65	−0.11	21.27	−0.59	400 (−39)
AZ91D	5	v_c_	5.23	−0.10	5.23	−0.10	6.08	−0.43	700 (−12)
AZ91D	30	v_c_	15.38	−0.27	14.01	0.04	17.66	−0.57	1200 (−42)
AZ31B	5	f_z_	18.38	−0.42	18.38	−0.42	31.89	−3.81	0.3 (+61)
AZ31B	30	f_z_	13.13	0.86	10.58	0.92	8.76	0.92	0.3 (−23)
AZ91D	5	f_z_	22.71	0.29	23.04	0.30	17.69	0.59	0.3 (−36)
AZ91D	30	f_z_	16.72	0.61	16.72	0.61	19.25	0.57	0.05 (+28)
AZ31B	5	a_p_	9.90	0.90	9.99	0.89	6.27	0.95	6 (−16)
AZ31B	30	a_p_	29.10	0.67	30.27	0.64	14.79	0.93	0.5 (−21)
AZ91D	5	a_p_	7.29	0.90	7.29	0.90	13.95	0.63	6 (−32)
AZ91D	30	a_p_	28.47	0.69	29.83	0.68	16.90	0.91	0.5 (+26)

**Table 9:** Maximum-likelihood hyperparameters of the Gaussian process models fitted to the complete curves.

Alloy	γ (°)	Parameter	n	ℓ	σf (°C)	σn (°C)	β0 (°C)	β1 (°C)
AZ31B	5	v_c_	9	0.12	0.004	4.0	315.3	3.9
AZ31B	5	f_z_	6	0.12	0.274	12.7	308.3	−11.8
AZ31B	5	a_p_	6	0.29	6.7	0.4	266.6	38.4
AZ31B	30	v_c_	9	0.26	16.2	0.2	264.0	9.8
AZ31B	30	f_z_	6	0.45	7.9	0.4	298.0	−45.7
AZ31B	30	a_p_	6	0.05	0.800	19.3	203.9	62.6
AZ91D	5	v_c_	9	2.93	0.003	5.4	281.8	3.1
AZ91D	5	f_z_	6	0.39	18.4	0.4	302.6	−30.7
AZ91D	5	a_p_	6	6.14	0.004	6.7	247.4	30.8
AZ91D	30	v_c_	9	2.04	0.005	12.1	259.5	−13.2
AZ91D	30	f_z_	6	8.32	0.007	11.8	276.2	−30.3
AZ91D	30	a_p_	6	0.54	22.6	8.6	171.4	59.8

## Data Availability

The original contributions presented in this study are included in the article. Further inquiries can be directed to the corresponding author.
